# IMPLEMENTATION OF A COMMUNITY WALKING PROGRAM FOR OLDER ADULTS WITH MOBILITY CHALLENGES—WALK-ON!

**DOI:** 10.1093/geroni/igad104.1693

**Published:** 2023-12-21

**Authors:** Barb Nicklas, Jaime Hughes, Kathryn Porter Starr

## Abstract

Clinical trials in controlled settings show walking interventions improve function and reduce mobility loss in older adults. In the NIA-funded LIFE study, participants with more compromised function derived the most benefit, yet improvements were not sustained. Based on this research, clinicians often prescribe walking for older patients. However, few communities provide accessible opportunities for those with limited function to engage in safe, ability-appropriate, walking. Additionally, no evidence-based National Council on Aging exercise programs emphasize ambulation over longer distances in supervised and social settings. In collaboration with community partners, we developed and initiated Walk On!, a group-based, facilitator-led program designed to improve walking endurance, balance, confidence, and social connectedness in older adults with mobility challenges. In addition to walking, the program incorporates balance, gait, and flexibility components, and follows a safe progression based on individual ability. Sessions take place for one-hour, twice/week, over 12 weeks. The program is currently offered at four sites in Winston-Salem and Charlotte, NC (two churches-one primarily black membership and one primarily white, a YWCA, and a community fitness center). This symposium provides an overview of Walk On! and initial implementation. Both quantitative and qualitative program data on feasibility, satisfaction, and effectiveness for improving physical and psychosocial outcomes will be presented. Based on these early data, we believe there are countless underserved older adults who may be socially isolated due to limited mobility, yet are willing and would benefit from incorporating more ambulation in a safe and accessible environment.

